# Elucidation of Motor Learning Mechanisms Based on Predictive Control and Self-Reflection in Single-Leg Drop-Jump Landings

**DOI:** 10.7759/cureus.83006

**Published:** 2025-04-25

**Authors:** Kota Maeda, Kohei Okuyama, Kazumasa Ukai, Takuma Tsuji, Yamaguchi Hideaki, Shigeki Yokoyama, Takayuki Kodama

**Affiliations:** 1 Physical Medicine and Rehabilitation, Graduate School of Health Sciences, Kyoto Tachibana University, Kyoto, JPN; 2 Physical Medicine and Rehabilitation, Kyoto Shimogamo Hospital, Kyoto, JPN; 3 Research, Kyoto Tachibana University, Kyoto, JPN

**Keywords:** bereitschaftspotential, error-related negativity, jump-landing movement, motor imagery, motor learning

## Abstract

Background*:*Jump-landing is a fundamental movement critical for enhancing athletic performance and preventing injuries, making the facilitation of rapid motor learning essential. Motor learning and performance are commonly evaluated using biomechanical measures. Although neurophysiological processes such as predictive control and self-reflection are thought to contribute to motor learning, studies from this perspective remain limited. In this study, we focused on three neural markers: Bereitschaftspotential (BP), which reflects predictive control before movement initiation; posterior parietal cortex (PPC) activity, which is involved in sensory information processing during motor learning; and error-related negativity (ERN), which reflects self-reflection following movement. We aimed to clarify the relationships between these neural markers and motor learning during jump-landing tasks.

Methods*:*A cross-sectional study was conducted with eight healthy male participants, each performing twenty single-leg drop jumps. Participants were instructed to land on a designated target point, and the error distance between the big toe and the target was measured. Reduction in error distance across trials was quantified as a learning curve, and its slope was used as an index of motor learning ability. Bereitschaftspotential (BP) was measured at the Cz electrode, and activity in the posterior parietal cortex (PPC) was analyzed at the Pz electrode; integral values over the three seconds prior to jump takeoff were calculated. ERN was extracted from the Fz electrode as the maximum negative amplitude occurring 50-150 ms after landing. Statistical analyses were conducted to examine the correlations between electroencephalography indices and the learning curve slope. In addition, classification using a support vector machine (SVM) was performed to assess whether ERN amplitude could predict high or low motor learning ability.

Results*:* BP and PPC activity were significantly negatively correlated with the learning curve slope, indicating faster motor learning. In contrast, ERN amplitude showed no significant correlation with the slope. However, the SVM classification model demonstrated that ERN amplitude could accurately predict high and low motor learning ability.

Conclusion*:* BP and PPC activity contributed to faster motor learning, while ERN enabled classification of learning ability. These findings suggest that predictive control, sensory integration, and self-reflection are key components of motor learning. This study is among the first to integratively examine the roles of BP, PPC, and ERN in a dynamic jump-landing. The findings demonstrate that predictive control, sensory integration, and self-reflection are key contributors to motor learning efficiency. These insights offer novel perspectives for assessment and training design in sports science and rehabilitation, with implications for performance enhancement and injury prevention.

## Introduction

In this study, ‘jump-landing’ refers to the full movement sequence from takeoff to landing, specifically involving single-leg drop-jump tasks. Jump-landing movements are often associated with sports-related injuries and are frequently observed in activities like volleyball and basketball [1]. These movements involve rapid deceleration and high impact during landing, making them a common focus in sports medicine and rehabilitation research [2]. Anterior cruciate ligament (ACL) injuries and ankle sprains frequently occur during the landing phase, making it essential to optimize movement strategies for injury prevention in physical therapy and athletic rehabilitation. Improper landing mechanics are a primary contributing factor to sports injuries, and motor learning during the landing phase is essential for rehabilitation [3]. The ability to acquire and refine landing mechanics efficiently plays a critical role in facilitating early return to sports and preventing re-injury.

Previous studies have primarily evaluated landing mechanics using biomechanical indicators, such as lower-limb joint angles and ground reaction forces (GRF). However, human movement is heavily influenced by the central nervous system, particularly brain function, which is essential for both motor performance and learning [4]. Therefore, in addition to biomechanical assessments, neurophysiological indicators, such as electroencephalography (EEG), should be considered when evaluating jump-related motor learning. Despite this, most studies have assessed motor learning solely based on performance outcomes, such as GRF or sport-specific skills [5,6], with limited investigation into its relationship with brain function, which is fundamental to the motor learning process.

Motor learning relies on internal models that simulate interactions between the body and the environment [7]. These models function through predictive control and feedback control. Predictive control generates motor commands in advance, while feedback control adjusts movement based on sensory input. Integrating these mechanisms improves learning efficiency and accuracy [8]. EEG markers such as Bereitschaftspotential (BP) and error-related negativity (ERN) reflect these processes: BP is linked to motor preparation and ERN to error detection, yet their roles in dynamic movements such as jump-landing remain underexplored.

Self-reflection is a key component of motor learning [9]. It enables individuals to evaluate performance after movement, identify errors, analyze their causes, and adjust subsequent movements accordingly. This process improves movement accuracy and adaptability by facilitating intuitive error detection and effective feedback processing. While predictive control supports pre-movement planning and execution, self-reflection may play a critical role in facilitating post-movement adaptation. The integration of predictive control, movement execution, and the process of self-reflection plays a central role in motor learning. Moreover, accurate processing of sensory input, including visual and proprioceptive signals, is essential for predictive control. The posterior parietal cortex (PPC) helps integrate this information to support motor planning. Previous studies suggest that precise sensory processing enhances internal model formation and facilitates motor learning [10,11].

BP and ERN are commonly used to assess predictive control and self-reflection in motor learning. BP is associated with movement planning and preparation, while ERN is related to self-reflection and error processing. We selected these markers because they directly correspond to the two core processes investigated in this study. BP reflects the brain’s preparatory state before movement onset, indicating central nervous system activity involved in motor planning and prediction. The amplitude of this potential has been shown to increase in response to enhanced motor preparation and motor imagery [12]. Moreover, BP activity increases as motor learning progresses, reflecting improvements in movement planning and the quality of preparation. BP originates primarily in the supplementary motor area (SMA) and plays a crucial role in both motor planning and predictive control [13]. In the context of sports and rehabilitation, BP is considered a key indicator of predictive control before movement execution, which has been suggested to contribute to injury prevention.

In contrast, ERN is a negative potential associated with error detection that arises following movement execution and is primarily observed in the anterior cingulate cortex (ACC) [14]. This potential emerges when an action deviates from the intended outcome and serves as a key marker of self-reflection and adaptive behavioral adjustment. ERN signals a neural process that rapidly detects intuitive errors and facilitates corrective adjustments in subsequent movements. Larger ERN amplitudes have been associated with heightened error sensitivity, thereby enhancing error-based learning and adaptive behavior [15].

Motor imagery (MI) has been reported to play an important role in motor learning. MI, the mental simulation of movement without actual execution, has been proposed as an effective strategy to enhance motor learning and prevent injuries. MI facilitates motor learning by activating neural circuits involved in motor control, even in the absence of physical movement [16].

Although EEG-based insights into motor learning are increasingly recognized, the relationship between BP, ERN, and sport-specific movements such as jump-landing remains unclear. Therefore, the aim of this study was to clarify the relationship between motor learning ability and EEG indices, specifically BP and ERN-during single-leg drop-jump landings. Based on this objective, we formulated the following hypothesis: motor learning in jump-landing tasks progresses more efficiently when BP and ERN activity levels are higher. Specifically, we hypothesized that participants with high BP amplitudes would exhibit optimal movement planning and predictive control before movement onset, whereas those with high ERN amplitudes would demonstrate effective error detection and self-reflection, leading to improved correction and adaptation during learning. Therefore, we propose that individuals exhibiting high levels of both BP and ERN engage in more effective predictive control and self-reflection processes, resulting in maximized learning accuracy and performance in jump-landing movements. This study is expected to provide a comprehensive understanding of the roles of predictive control and self-reflection in jump-landing movement learning, offering new insights into motor learning through EEG markers.

## Materials and methods

Participants

Eight healthy adult male volunteers aged 20-30 years participated in the study and were compensated for their participation. The exclusion criteria were as follows: (i) current lower limb pain or orthopedic disorders, (ii) history of lower limb surgery, (iii) central nervous system or psychiatric disorders, (iv) insufficient sleep, (v) visual impairment (corrected vision with glasses or contact lenses was permitted), and (vi) caffeine consumption on the day of the experiment.

All participants received verbal and written explanations of the study’s purpose, procedures, and content and provided written informed consent. The Ethics Review Committee of Kyoto Tachibana University approved the study (approval number: 23-48).

Experimental task

**Figure 1:**
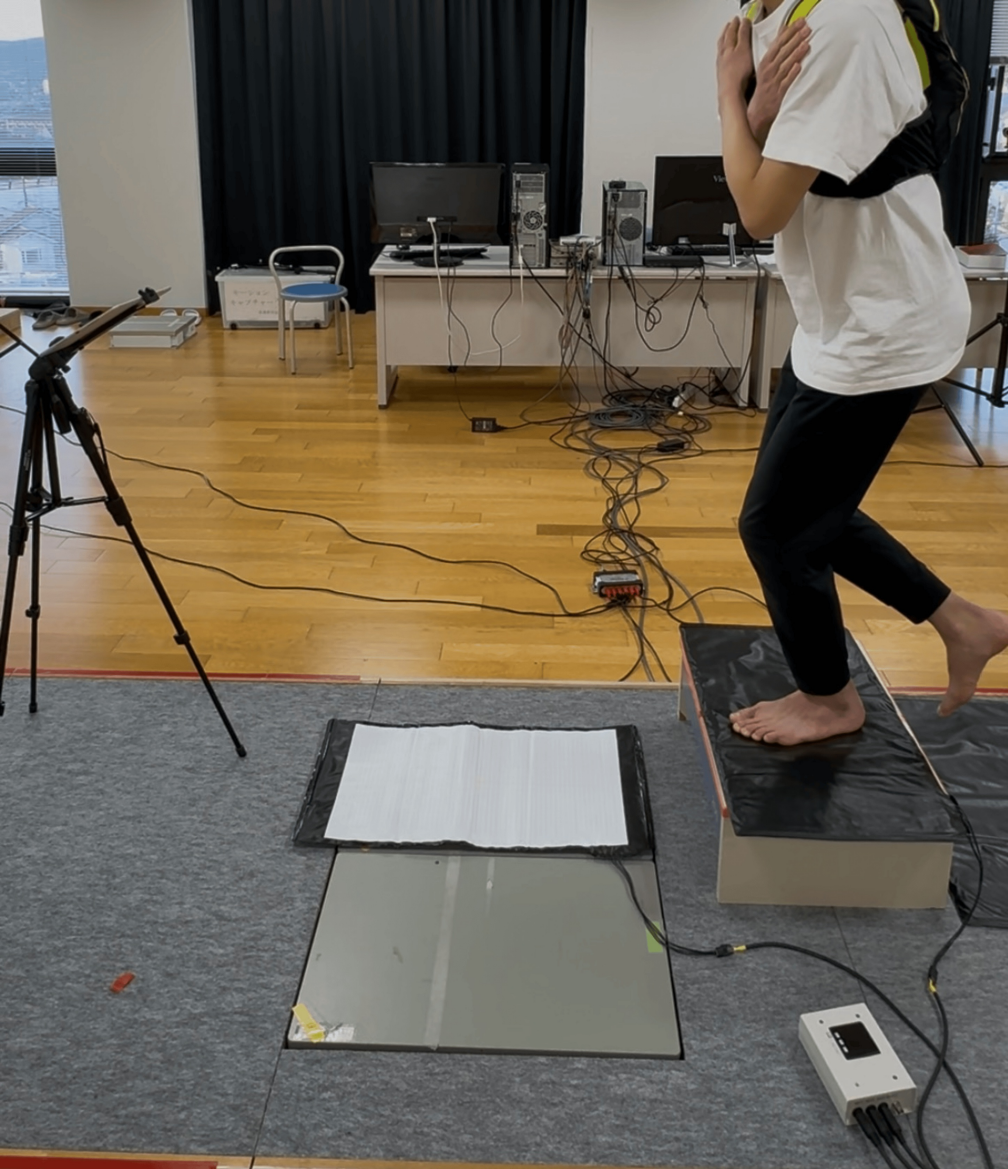
Overview of the experimental setup

The motor task involved performing 20 single-leg drop jumps using the dominant leg, defined as the foot used to kick a ball. Participants stood on a 20 cm-high platform and were instructed to land on a circular marker (diameter: 1 cm) positioned 45 cm in front of the platform. The instruction provided was, "Land so that the tip of your big toe touches the marker." The examiner emphasized that toe-marker contact was required to standardize movement execution and effectively elicit the ERN. The procedure was as follows: Upon the cue "Stand on the platform," the participant stood in position. At "Prepare," they raised the non-dominant leg and prepared to jump. After visually confirming the target marker, participants jumped upon hearing the "Hai" signal, which was given after a 3-s delay. Participants remained still for 5 s post-landing. A 30-s rest period was provided between jumps, with a 3-min rest after every 10 jumps. This sequence is illustrated in Figure 1.

GRF analysis

GRF during landing was measured using a force plate (Anima Corp., Tokyo, Japan). The vertical component of the GRF was analyzed to assess shock absorption. A higher peak vertical GRF indicates greater mechanical loading on the lower limbs, which is associated with an increased risk of ACL injury and ankle sprains. The shock absorption coefficient was calculated by dividing the peak vertical GRF by the time required to reach the peak (ms). A higher coefficient indicates lower shock absorption capacity, whereas a lower coefficient reflects a more stable landing [17].

EEG measurement and analysis

EEG was recorded using a Polymate VAP5148 biosignal amplifier (Miyuki Giken Co., Ltd., Tokyo, Japan) following the international 10-10 electrode placement system, with a sampling rate of 1 kHz and 28 channels. The trigger for BP was defined as the moment the foot left the 20-cm platform, while the ERN trigger was defined as the moment of landing. Foot-off and foot-on timings were detected using a foot pressure sensor (CARETECH Plus, Aichi, Japan) and synchronized with EEG data. EEG denoising and analysis were conducted using EMSE software (Miyuki Giken Co.) and EEGLAB (Swartz Center for Computational Neuroscience, University of California San Diego, La Jolla, CA) in MATLAB R2024a (MathWorks, Natick, MA). Foot-off and foot-on triggers were first extracted using EMSE and then imported into EEGLAB for analysis. A 6-35-Hz bandpass filter was applied, followed by independent component analysis to remove noise components.

For BP analysis, electrodes Fz, Cz, and Pz, which are associated with predictive control, were used. EEG data from 3 s before foot-off were extracted, and because BP is a time-integrated measure, the integral value over this 3-s interval was calculated [18]. The same procedure was applied to Cz and Pz, and the mean across 20 trials was used for further analysis. For ERN analysis, the Fz electrode was used. The peak negative amplitude between 50 and 150 ms post-landing was extracted, visually confirmed to fall within this range, and averaged across 20 trials.

Motor learning ability evaluation

Motor learning ability was evaluated based on the error distance, defined as the distance between the tip of the big toe and the target marker at landing. This metric accurately reflected the participant’s ability to predict and control the jump trajectory. Landing positions were recorded using an iPad (Apple, Cupertino, CA) positioned to clearly capture each landing. The recorded videos were analyzed using ImageJ software (National Institutes of Health, Bethesda, MD) to calculate error distances.

The center of the target marker was designated as the origin (0,0), with lateral and vertical deviations measured along the x- and y-axes, respectively. The coordinates of the toe and marker were extracted, and the error distance was calculated using the Pythagorean theorem. A smaller error distance indicated greater landing accuracy. Motor learning improvements were assessed based on changes in error distance across repeated trials.

Learning curve analysis

Error distance for each trial was plotted against the number of trials to generate a learning curve, which was fitted using a linear regression model (y = ax + b) [19]. The slope (a) represented the rate of decrease in error distance, with a greater absolute slope indicating faster improvement in landing accuracy. The intercept (b) corresponded to the error distance in the first trial. Participants were classified into high-learning-ability and low-learning-ability groups based on the median slope value. This median-based classification ensured robustness against outliers and facilitated stable grouping [20]. The classification was subsequently used in statistical analyses to assess individual differences in motor learning ability.

Statistical analysis

The Shapiro-Wilk test was used to assess data normality. After confirming normality, Spearman’s rank correlation coefficient was used to evaluate relationships between jump-landing movements and neurophysiological indicators. Statistical analyses were performed using IBM SPSS Statistics for Windows, Version 28 (Released 2021; IBM Corp., Armonk, New York, United States), with significance set at p < 0.05.

To predict motor learning ability from ERN amplitude values, a machine learning model was constructed using a support vector machine (SVM) in MATLAB. SVM is an efficient classification method that performs well with small datasets due to its high generalization performance [21]. It was selected over regression analysis because this study aimed to classify motor learning ability into high and low groups. Although regression is appropriate for predicting continuous outcomes, SVM is better suited for binary classification.

To enhance SVM model accuracy, the sample size was increased using the bootstrap method, which improves estimation accuracy by generating pseudo-datasets through random resampling of the original dataset [22]. Using this approach, the sample size was increased to 30. The number of bootstrap samples was determined based on Monte Carlo cross-validation [23].

SVM model performance was evaluated using four metrics: accuracy, precision, recall, and F1 score. Accuracy reflected the proportion of correct predictions, precision represented the proportion of correctly predicted positive cases, recall indicated the proportion of actual positive cases correctly identified, and the F1 score, the harmonic mean of precision and recall, assessed the balance between the two. A score of ≥0.7 was considered indicative of acceptable model performance, while a score of ≥0.8 was interpreted as very good performance [24].

## Results

**Figure 2:**
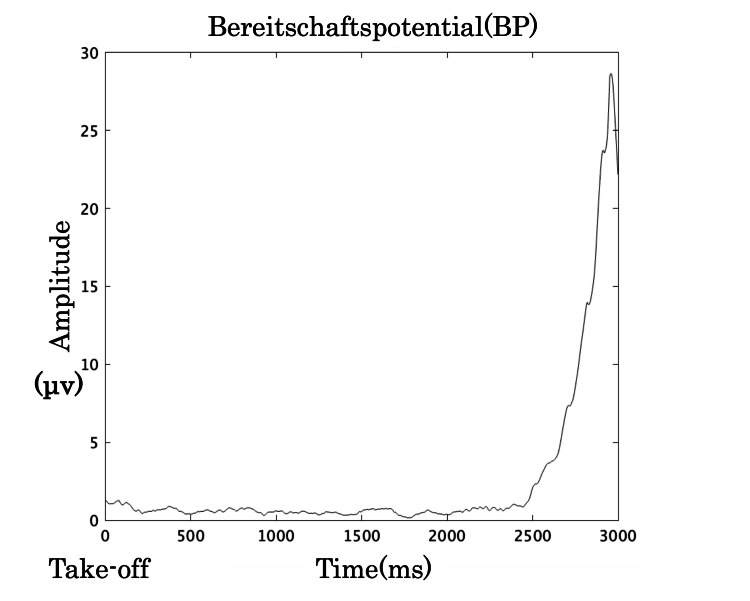
BP waveform after noise processing

**Figure 3:**
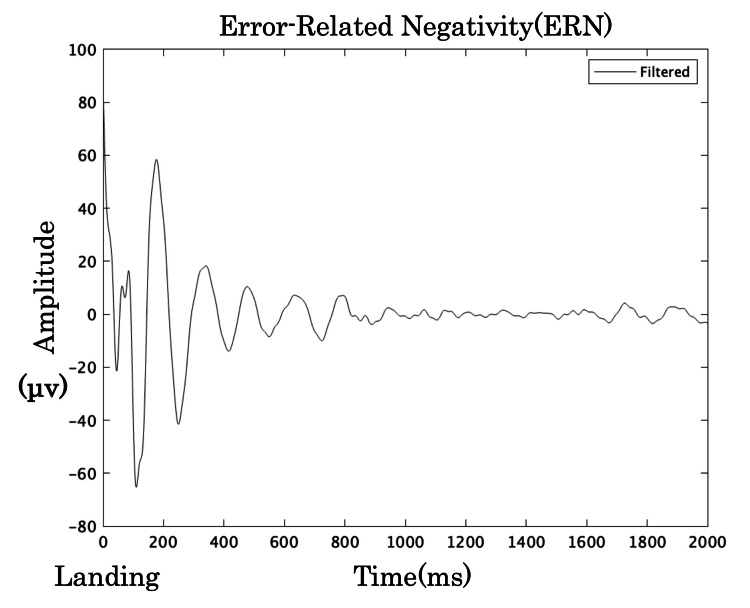
ERN waveform after noise processing

**Figure 4:**
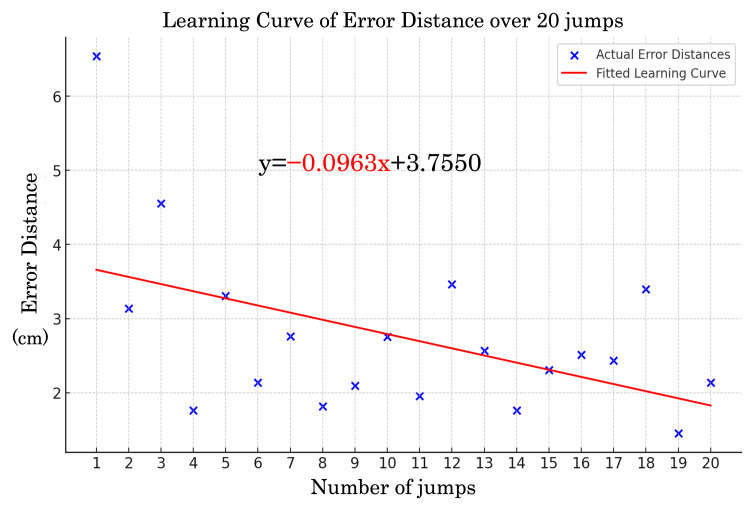
Learning curve of error distance over 20 jump trials

The results for neurophysiological indicators, motor learning ability, and GRF are summarized in Table 1. EEG noise processing effectively extracted clear BP and ERN waveforms, as shown in Figures 2, 3. The learning curve, derived from the reduction in error distance across trials, is illustrated in Figure 4.

**Table 1:** Motor learning ability, neurophysiological indicators, and ground reaction force in jump-landing movement ERN: Error-related negativity; BP: Bereitschaftspotential

Target	Classifying	Slope of the learning curve	ERN	BP (Cz)	Fz	Pz	Vertical component	Loading rate
A	1	-0.245	-288.3	3273.2	2548.6	966.1	2565.1	61.6
B	1	-0.205	-109.7	4540.7	4072.1	1626.9	3223.8	94.4
C	1	-0.094	-62.3	732.0	412.6	647.3	3105.2	77.7
D	0	-0.056	-20.0	1077.2	1249.8	392.7	2155.3	71.6
E	0	-0.052	-93.6	671.1	577.3	191.1	2697.6	88.2
F	1	-0.125	-106.9	3121.8	1229.6	276.6	3016.1	147.3
G	0	-0.056	-115.5	752.9	531.7	434.6	2361.1	86.2
H	0	-0.074	-122.3	834.4	495.1	901.4	2324.2	60.1

Correlation between jump-landing movement and neurophysiological indicators

A strong negative correlation was observed between the slope of the learning curve and BP (r = -0.762, p < 0.01). Similarly, the slope of the learning curve showed a strong negative correlation with PPC activity (r = -0.714, p < 0.01). Additionally, a strong positive correlation was found between BP and Fz (r = 0.810, p < 0.01). However, no significant correlations were observed between GRF parameters and any of the neurophysiological markers, including BP, ERN, and PPC activity. The correlation results are presented in Table 2. 

**Table 2:** Correlation between jump-landing movement and neurophysiological indicators ERN: Error-related negativity; BP: Bereitschaftspotential

	Slope of the learning curve	ERN	BP (Cz)	Fz	Pz	Vertical component	Loading rate
ERN	0.524	1.000	-0.381	-0.143	-0.571	0.095	0.310
Slope of the learning curve	1.000	0.524	-0.762*	-0.429	-0.714*	-0.500	-0.048
BP (Cz)	-0.762*	-0.381	1.000	0.810*	0.595	0.167	0.095
Fz	-0.429	-0.143	0.810*	1.000	0.286	0.143	0.095
Pz	-0.714*	-0.571	0.595	0.286	1.000	0.214	0.262
Vertical component	-0.500	0.095	0.167	0.143	0.214	1.000	-0.357
Loading rate	-0.048	0.310	0.095	0.262	-0.357	0.667	0.667
*Significant at p < 0.05 (two-tailed).							

Classification of motor learning ability using SVM

**Figure 5:**
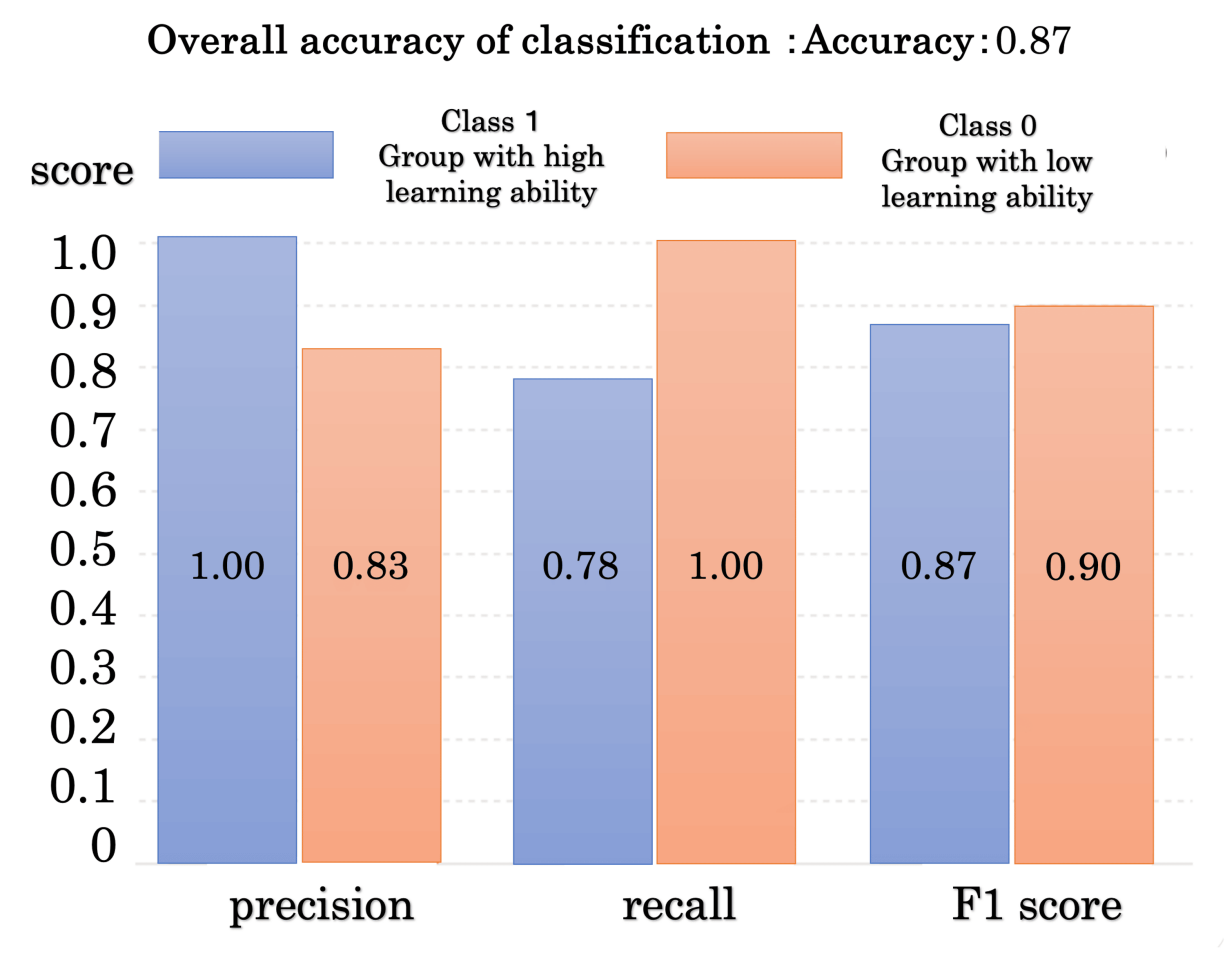
Classification performance results of the SVM SVM: Support vector machine

Motor learning ability was classified as high or low based on ERN amplitude values. The overall classification accuracy was 0.87. For Class 1 (high learning ability), precision was 1.00, recall was 0.78, and the F1 score was 0.87. For Class 0 (low learning ability), precision was 0.83, recall was 1.00, and the F1 score was 0.90. These results are summarized in Figure 5.

## Discussion

Considerations regarding BP

In this study, a significant negative correlation was observed between the integral value of BP and the slope of the learning curve, indicating that participants with higher BP activity exhibited faster motor learning in jump-landing movements.

BP reflects neural activity preceding movement onset and is thought to enhance motor planning quality. Previous studies have reported that BP facilitates the formation of internal models via the SMA and contributes to generating appropriate motor output [8,13]. The SMA, recognized as the primary source of BP, provides the neural basis for motor planning. Additionally, BP has been observed during MI and is believed to be associated with SMA activation [12].

Participants with higher BP activity may have exhibited more robust internal model formation via the SMA, leading to improved motor planning quality. This enhancement could have contributed to better predictive control, reduced movement error, and greater motor learning efficiency. Furthermore, MI is considered a crucial factor in enhancing athletic performance, and previous studies have shown that BP is elicited during MI [12]. MI activates the SMA and premotor cortex, and in this study, participants with higher BP activity also demonstrated greater learning efficiency. These findings suggest that pre-movement MI may enhance predictive control by improving motor planning quality.

Considerations regarding PPC

This study found a significant negative correlation between the integral value of PPC and the slope of the learning curve, indicating that participants with higher PPC activity exhibited faster motor learning during jump-landing movements.

The PPC integrates visual and proprioceptive information and plays a key role in adaptive motor planning [25]. Buneo et al. [26] reported that the PPC integrates information about target and limb positions, contributing to the generation of appropriate motor plans. Additionally, the PPC has been shown to improve spatial perception accuracy by integrating visually predicted information based on self-motion with sensory feedback [27].

The negative correlation between PPC activity and the slope of the learning curve observed in this study suggests that PPC activity before movement execution may facilitate motor learning by enhancing the integration of visual and proprioceptive information. Participants needed to visually identify the target and integrate proprioceptive input from the joints and muscles of the lower limbs before initiating the jump. Those with higher PPC activity may have achieved more effective sensory integration, thereby improving motor learning efficiency.

These findings align with previous reports, suggesting that higher PPC activity may contribute to more efficient motor learning by supporting sensory integration before movement execution and feedback processing afterward.

Considerations regarding ERN

This study used an SVM to classify participants based on ERN amplitude to predict motor learning ability. The results showed a classification accuracy of 87%, indicating that participants with high and low learning abilities could be distinguished (Figure 4). Furthermore, participants with higher ERN amplitudes were more likely to be classified into groups with steeper learning curve slopes, suggesting faster motor learning.

The ERN is an event-related potential that reflects unconscious error detection after movement execution and primarily originates from the ACC [14]. The ACC plays a central role in rapidly processing error-related information and adjusting adaptive behavior accordingly. Previous studies have reported that greater ERN amplitudes are associated with enhanced error detection and learning progression [28]. Increased ERN amplitude has also been suggested to facilitate quicker error recognition, contributing to more efficient motor learning [29].

The results of this study align with previous findings [29], supporting the role of ERN in signaling error-related information and facilitating adaptive behavioral modification. Furthermore, this study was conducted in an environment that relied solely on intrinsic feedback, without extrinsic feedback. Under these conditions, participants with higher ERN amplitudes may have progressed more efficiently in motor learning through self-reflection. These findings further support previous studies, indicating that ERN amplitude is associated with enhanced error detection and motor learning progression. Collectively, these results suggest that ERN serves as a neural mechanism for error detection and adaptive correction, thereby contributing to motor learning efficiency.

Integrated contribution of BP, PPC, and ERN across stages of motor learning

The results of this study suggest that BP, PPC, and ERN contribute to different but complementary stages of motor learning, collectively supporting improved learning efficiency. Specifically, BP is associated with predictive control prior to movement execution, PPC reflects sensory integration during motor planning, and ERN is related to error monitoring and self-reflection after movement. Figure 5 summarizes these processes, predictive control, sensory information processing, and self-reflection, and highlights how their integration may facilitate efficient motor learning in dynamic tasks such as jump-landing.

**Figure 6:**
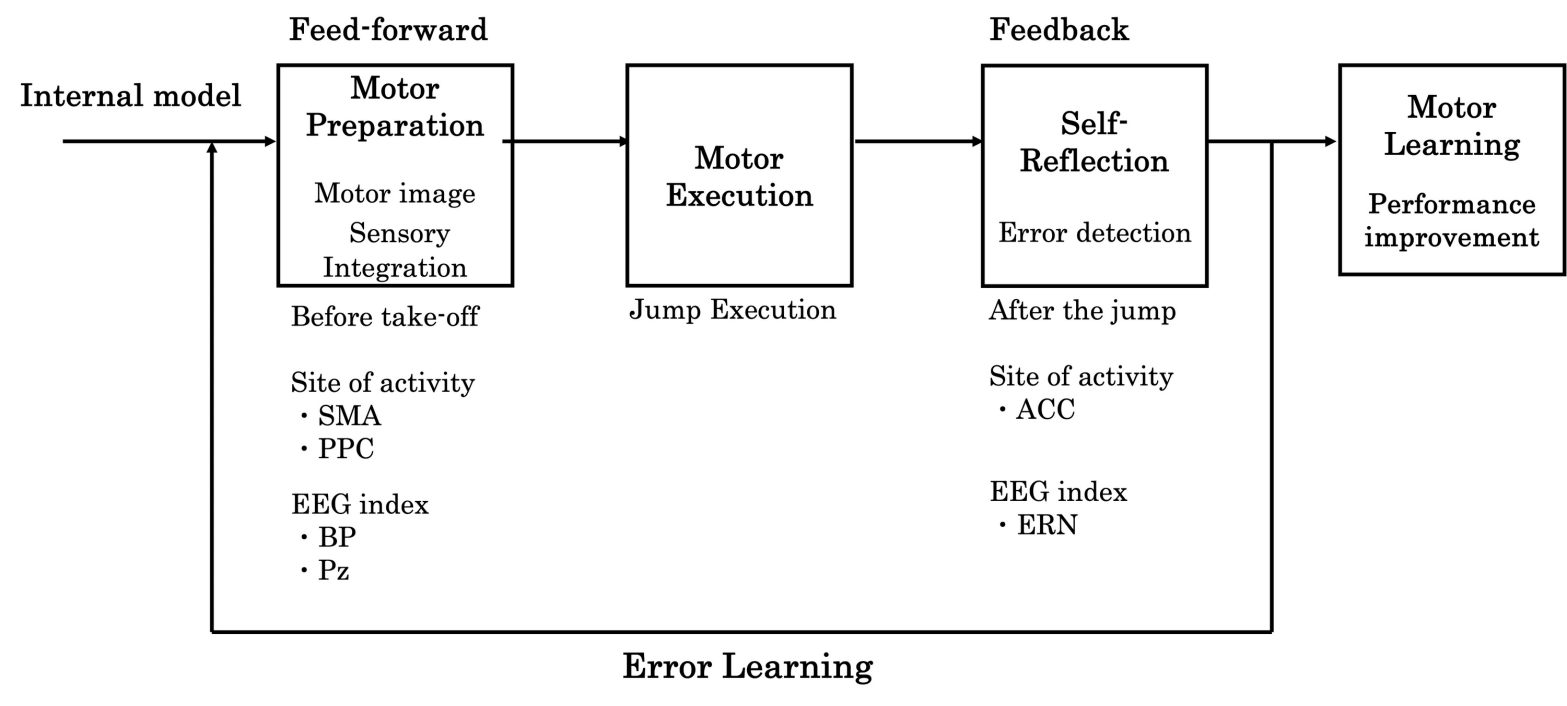
Integrated flow of predictive control, sensory processing, and self-reflection in motor learning SMA: Supplementary motor area; PPC: posterior parietal cortex; ERN: error-related negativity; ACC: anterior cingulate cortex; BP: Bereitschaftspotential

Limitations

This study has several limitations. First, the relatively small sample size may limit the generalizability of the findings. This issue is particularly relevant for machine learning analysis using an SVM, where sample size directly influences model accuracy and generalization performance [21]. Although a bootstrap method was applied to enhance reliability, potential dependence on a specific dataset cannot be ruled out. Future studies should focus on improving model reproducibility and generalizability through large-scale data collection, multicenter collaboration, and international data sharing.

Second, only male participants were included. Previous research suggests that sex differences may influence BP and ERN characteristics, as well as motor learning processes. For example, women may be more sensitive to error detection and emotional factors [30]. Future studies should include female participants to examine how these differences affect motor learning.

Third, the biomechanical perspective of this study was limited. While GRF was analyzed, detailed data on muscle activity and joint kinematics were not collected. Integrating neurophysiological and biomechanical indicators could provide a more comprehensive understanding of the relationships among motor learning mechanisms, brain function, and physical movement. Future studies incorporating motion capture systems and electromyography may further elucidate the roles of predictive and feedback controls during jump landings.

## Conclusions

This study evaluated the neural basis of motor learning during jump landings using EEG indicators. The results revealed that BP, associated with predictive control; PPC, involved in sensory information integration; and ERN, related to self-reflection, were significantly linked to motor learning progression in the jump-landing task. Additionally, the application of ICA and SVM enhanced EEG analysis precision and improved the classification of motor learning ability, providing a neurophysiological demonstration of the relationship between brain activity and motor learning ability. This study is the first to investigate motor learning during jump landings using EEG, contributing to a deeper understanding of the neural mechanisms underlying dynamic sports movements. These findings suggest broader applicability to other motor tasks and real-time feedback systems, offering new perspectives for optimizing training and rehabilitation.
